# Infant Homicides Within the Context of Safe Haven Laws — United States, 2008–2017

**DOI:** 10.15585/mmwr.mm6939a1

**Published:** 2020-10-02

**Authors:** Rebecca F. Wilson, Joanne Klevens, Dionne Williams, Likang Xu

**Affiliations:** ^1^Division of Violence Prevention, National Center for Injury Prevention and Control, CDC; ^2^Division of Injury Prevention, National Center for Injury Prevention and Control, CDC.

Homicide is the 13th leading cause of death among infants (i.e., children aged <1 year) in the United States (*1*). Infant homicides occurring within the first 24 hours of life (i.e., neonaticide) are primarily perpetrated by the mother, who might be of young age, unmarried, have lower educational attainment, and is most likely associated with concealment of an unintended pregnancy and nonhospital birthing (*2*). After the first day of life, infant homicides might be associated with other factors (e.g., child abuse and neglect or caregiver frustration) (*2*). A 2002 study of the age variation in homicide risk in U.S. infants during 1989–1998 found that the overall infant homicide rate was 8.3 per 100,000 person-years, and on the first day of life was 222.2 per 100,000 person-years, a homicide rate at least 10 times greater than that for any other time of life (*3*). Because of this period of heightened risk, by 2008 all 50 states* and Puerto Rico had enacted Safe Haven Laws. These laws allow a parent^†^ to legally surrender an infant who might otherwise be abandoned or endangered (*4*). CDC analyzed infant homicides in the United States during 2008–2017 to determine whether rates changed after nationwide implementation of Safe Haven Laws, and to examine the association between infant homicide rates and state-specific Safe Haven age limits. During 2008–2017, the overall infant homicide rate was 7.2 per 100,000 person-years, and on the first day of life was 74.0 per 100,000 person-years, representing a 66.7% decrease from 1989–1998. However, the homicide rate on first day of life was still 5.4 times higher than that for any other time in life. No obvious association was found between infant homicide rates and Safe Haven age limits. States are encouraged to evaluate the effectiveness of their Safe Haven Laws and other prevention strategies to ensure they are achieving the intended benefits of preventing infant homicides. Programs and policies that strengthen economic supports, provide affordable childcare, and enhance and improve skills for young parents might contribute to the prevention of infant homicides.

Since 1999, when Texas became the first state to implement Safe Haven Laws, an estimated 4,100 infants have been safely surrendered nationwide (*5*). Safe Haven Laws are applied differently in each state, and one notable difference is the age limit of legal relinquishment (*4*). For example, 11 states and Puerto Rico limit relinquishment to infants who are aged ≤3 days, whereas 19 states allow relinquishment up to age 1 month (*4*). North Dakota allows relinquishment of infants aged <1 year (*4*).

Data for this analysis come from the National Vital Statistics System,**^§^** which includes a linked birth and death certificate for >99% of infants who die in the United States. Birth certificates provided demographic characteristics present at birth (e.g., mother’s age). Death certificates indicated both an underlying cause and manner of death, which the medical examiner or coroner is primarily responsible for certifying. Infant homicide was defined as the death of a child before the first birthday, using the *International Classification of Diseases, Tenth Revision* (ICD-10) underlying cause of death codes X85–Y09, Y87.1, U01, and U02.^¶^ Age at death was calculated as the difference in days between the dates of birth and death recorded on the death certificate; an infant killed on their date of birth had an age at death of 0 days. To examine the association between homicide rates and state-specific Safe Haven age limits for legal relinquishment, infant homicides were categorized using age limits specified in state Safe Haven Laws as of 2016** (*4*). These age limits were treated as stable and applied throughout the entire study period. Data years 2008–2017 were used to coincide with national enactment and implementation of Safe Haven Laws. Homicide rates were presented as rates per person-years of exposure, which allowed for the calculation of homicide risk by age of infant, because infant homicides occurred at different times during infancy (e.g., day of birth, week one).^††^

During 2008–2017, the U.S. population aged <1 year accounted for 39,984,337 person-years of exposure; days of birth accounted for 109,471 person-years (0.27%). The remainder of infancy accounted for 39,874,866 person-years. An estimated 2,851 infants were victims of homicide during 2008–2017 (Table 1). The overall infant homicide rate was 7.2 per 100,000 person-years. The homicide rate of infants killed on the day of birth was 74.0 per 100,000 person-years, which was 5.4 times higher than the rate at any other time of life (Supplementary Table, https://stacks.cdc.gov/view/cdc/93750).

**TABLE 1:** Number,* percentage,^†^ and rate^§^ of infant homicides (N = 2,851), by demographic characteristics — restricted-use National Vital Statistics System linked birth and infant death data, United States,^¶^ 2008–2017

Characteristic	No. (%) of homicides^†^	Rate^§^ (95% CI)	p-value
**Age of infant**			
All aged <1 year**	2,851	7.2 (6.9-7.4)	N/A
First day of life	81 (2.8)	74.0 (58.8-92.0)	N/A
**Sex of infant**			
Male	1,636 (57.4)	8.0 (7.6-8.4)	<0.001
Female	1,215 (42.6)	6.2 (5.9-6.6)	Referent
**Mother’s age group (yrs)**			
<20	565 (19.8)	18.7 (17.1-20.2)	<0.001
20–29	1,860 (65.2)	9.1 (8.7-9.5)	<0.001
≥30	426 (14.9)	2.6 (2.3-2.8)	Referent
**Mother’s race/ethnicity** ^††^			
White, non-Hispanic	1,771 (62.1)	5.9 (5.6-6.1)	Referent
Black, non-Hispanic	929 (32.6)	14.4 (13.5-15.4)	<0.001
AI/AN, non-Hispanic	68 (2.4)	14.9 (11.6-18.9)	<0.001
Asian/Pacific Islander, non-Hispanic	83 (2.9)	3.1 (2.4-3.8)	<0.001
**Mother’s marital status**			
Married	705 (24.7)	3.0 (2.8-3.2)	Referent
Unmarried	2,137 (75.0)	13.4 (12.8-14.0)	<0.001
Unknown	9 (0.3)	—	—
**Mother’s highest educational level**			
Less than HS	698 (24.5)	12.2 (11.3-13.1)	Referent
HS or GED certificate	939 (32.9)	10.8 (10.1-11.5)	0.016
Some college, no degree	504 (17.7)	7.1 (6.5-7.7)	<0.001
Associate or bachelors’ degree	193 (6.8)	2.1 (1.8-2.4)	<0.001
Graduate degree	37 (1.3)	1.0 (0.7-1.4)	<0.001
Unknown	480 (16.8)	—	
**Infant’s place of birth**			
Hospital	2,730 (95.8)	7.0 (6.7-7.2)	Referent
Freestanding birth center	5 (0.2)	—	—
Residence	82 (2.9)	23.7 (18.9-29.5)	<0.001
Other location	20 (0.7)	66.9 (40.9-103.3)	<0.001
Unknown	14 (0.5)	—	—

**Abbreviations:** AI/AN = American Indian/Alaska Native; CI = confidence interval; GED = General Education Development; HS = high school; N/A = not applicable.

* During 2008–2017, approximately 2,919 infants were victims of homicide (https://webappa.cdc.gov/sasweb/ncipc/mortrate.html). Because this study used restricted-use National Vital Statistics System linked birth and infant death data, 68 infant homicides were excluded because the corresponding birth and death certificates could not be linked.

^†^ Percentages might not sum to 100% because of rounding.

^§^ Number of deaths per 100,000 person-years. Rates are not reported for subgroups in which the number of infant homicides is <20 or response is unknown.

^¶^ Infant homicides for Puerto Rico were not available for this analysis.

** Includes infants who died on first day of life.

^††^ Mother’s race/ethnicity is the best measure of race/ethnicity of the infant; thus, infant race/ethnicity is based on mother’s race/ethnicity as reported on the infant’s birth certificate.

The rate among males (8.0), who accounted for 57.4% of infant homicides, was slightly higher than that among females (6.2) (Table 1). Infants of non-Hispanic White (White) mothers accounted for 62.1% of homicides; however, rates among infants of non-Hispanic Black (Black) mothers (14.4), and non-Hispanic American Indian/Alaska Native (AI/AN) mothers (14.9) were more than twice the rate among infants of White mothers (5.9). Infants of Asian/Pacific Islander mothers had the lowest homicide rate (3.1). In addition, although infants of mothers aged 20–29 years accounted for almost two thirds (65.2%) of infant homicides, the rate among infants of mothers aged <20 years (18.7) was more than twice that among infants of mothers aged 20–29 years (9.1) and more than seven times that among infants of mothers aged ≥30 years (2.6).

Overall, 75.0% of infant homicide victims were born to unmarried mothers; the homicide rate among these infants (13.4) was approximately 4.5 times the rate per 100,000 person-years among infants born to married mothers (3.0). Nearly all infant homicide victims were born at a hospital (95.8%); however, among the small percentage who were born at a residence (2.9%) or another location (0.7%), the homicide rates (23.7 and 66.9) were approximately 3.4 and 9.6 times the rate among infants born at a hospital. Moreover, in the 2,371 cases where the mother’s education level was reported (83.2% of all infant homicides), homicide rates were higher among infants of mothers with lower education levels (less than high school, 12.2; high school graduation or equivalent, 10.8) than among infants whose mothers had higher education levels (1.0–7.1).

The percentage of homicides occurring each week of infancy varied (Figure). The first peak occurred in the first week of life, when 3.9% of all homicides occurred. A second peak occurred at week 11. Among the 111 infant homicides that occurred during the first week of life during 2008–2017, 73.0% occurred within the first 24 hours of life, and approximately two thirds of those infants (65.4%) were born at a residence.

**FIGURE:**
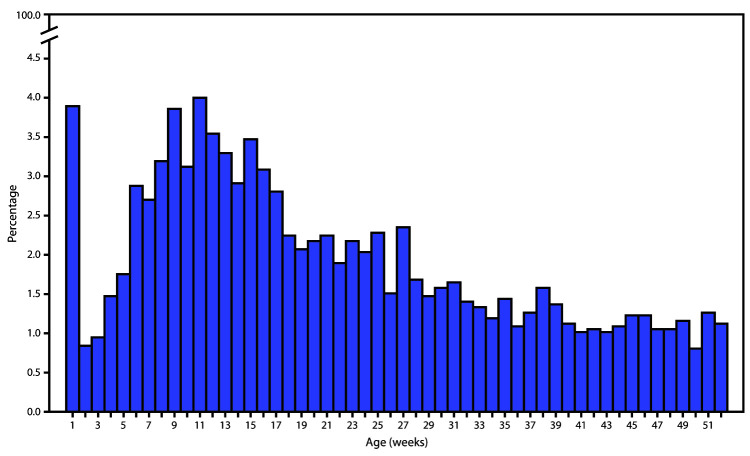
Percentage of infant homicides, by age at death (weeks) — restricted-use National Vital Statistics System, linked birth and infant death data, United States, 2008–2017

Most (92.4%) homicides occurred among infants who were too old for Safe Haven relinquishment at the time of their deaths; however, there was no obvious association between infant homicide rates and Safe Haven age limits (Table 2). For example, the infant homicide rates in states that limit relinquishment to ≤7 days and ≤14 days were 7.0 and 9.4 per 100,000 person-years, respectively. Conversely, the infant homicide rate for states that limit relinquishment to ≤45 days compared with ≤60 days was 10.6 and 7.3, respectively.

**TABLE 2:** Number,* percentage,^†^ and rate^§^ of homicides among infants (N = 2,849), by state^¶^ and corresponding Safe Haven Law age limit category — restricted-use National Vital Statistics System linked birth and infant death data, United States, 2008–2017

State/Area where homicide occurred	Safe Haven Law age limit	No. (%) of homicides^†^	Rate per 100,000 person-years (95% CI)^§^
**Alabama, Arizona, California, Colorado, Hawaii, Michigan, Mississippi, Tennessee, Utah, Washington, Wisconsin**	**3 days**	**738 (25.9)**	**6.3 (5.8–6.7)**
Florida, Georgia, Massachusetts, Minnesota, New Hampshire, North Carolina, Oklahoma	7 days	478 (16.8)	7.0 (6.4–7.6)
Maryland	10 days	54 (1.9)	7.7 (5.7–10.0)
Delaware, District of Columbia, Iowa, Virginia, Wyoming	14 days	162 (5.7)	9.4 (8.0–10.9)
Alaska	21 days	—	—
Arkansas, Connecticut, Idaho, Illinois, Indiana, Kentucky, Louisiana, Maine, Montana, Nebraska, Nevada, New Jersey, New York, Ohio, Oregon, Pennsylvania, Rhode Island, Vermont, West Virginia	30 days	923 (32.4)	7.4 (6.9–7.8)
Kansas, Missouri	45 days	124 (4.4)	10.6 (8.7–12.4)
South Carolina, South Dakota, Texas	60 days	335 (11.8)	7.3 (6.5–8.0)
New Mexico	90 days	22 (0.8)	8.6 (5.4–13.0)
North Dakota	<1 year	—	—

**Abbreviation:** CI = confidence interval.

* During 2008–2017, approximately 2,919 infants were victims of homicide https://webappa.cdc.gov/sasweb/ncipc/mortrate.html. Because this study used restricted-use National Vital Statistics System linked birth and infant death data, 68 infant homicides were excluded because the corresponding birth and death certificates could not be linked. The District of Columbia did not enact a Safe Haven Law until 2009; therefore, the two infant homicides that occurred in 2008, in the District of Columbia, were removed when infant homicide rates were examined within the context of Safe Haven Laws. Counts are not reported when the number of infant homicides is <10.

^†^ Percentages might not sum to 100% because of rounding.

^§^ Infant homicide rates are based on the state in which the infant’s death occurred (i.e., state of occurrence). Rates are not reported when number of infant homicides is <20. Denominator includes number of live births multiplied by the Safe Haven days in each Safe Haven age limit category.

^¶^ Infant homicides for Puerto Rico, which has a Safe Haven Law age limit of 3 days, were not available for this analysis.

## Discussion

In this study, the overall infant homicide rate (7.2 per 100,000 person-years) represented a 13.3% decrease from the 8.3 rate reported during 1989–1998 (*3*). Maternal characteristics associated with infant homicide included young age, being unmarried, having lower educational attainment, having a nonhospital birthing, Black race, and AI/AN ethnicity.

Among infants, the highest risk for homicide is on the day of birth. The rate on the day of birth in this study (74.0 per 100,000 person-years) represented a 66.7% decrease from the rate of 222.2 during 1989–1998 (*3*), but the rate on day of birth was still at least 5.4 times higher than the rate at any other time during life. Infant homicides occurring on the day of birth are primarily perpetrated by young, unmarried mothers with lower education levels who do not seek prenatal care; these homicides often are associated with concealment of an unintended pregnancy, and giving birth at a residence (*2*). After the first day of life, an infant homicide might occur within the context of young parental age, caregiver frustration, maternal mental illness, removal of an unwanted child, or abuse or neglect; depending on the context, the homicide might be perpetrated by the mother (*2*), mother’s male companion, or the biologic father of the infant (*6*). The presence or absence of these factors is important when assessing safety and quality of the infant’s home environment. Racial disparities in infant homicides might be attributed, at least in part, to the fact that Black and AI/AN families are more likely to experience sociodemographic disparities and poverty compared with White families (*7*). Circumstances of poverty (e.g., inadequate resources for childcare, housing, and food) might make parenting difficult (*7*). In addition, the association between infant homicide and Safe Haven age limits did not follow a linear pattern of risk, suggesting that rates cannot be explained by Safe Haven age limits, but might be related to other factors (e.g., maternal age or unintended pregnancy) (*2*). Given that most (92.4%) homicides occurred among infants who were too old for Safe Haven relinquishment at the time of their deaths, states are encouraged to evaluate the effectiveness of their Safe Haven Laws and other prevention strategies to ensure they are achieving the intended benefits of preventing infant homicides.

The findings in this report are subject to at least two limitations. First, an infant’s death might be misclassified on the death certificate (*8*) or undiscovered, leading to potential underascertainment or overascertainment of infant homicides. The lack of precise pathological markers for live births or cause of death can lead to errors in coding of the manner of death (*9*). Second, homicide rates for Safe Haven age-limit categories were calculated using age limits specified in state statutes as of December 2016. Two changes were made to state-specific age limits; one occurred during the study period and one after. In both instances, the age limit was raised to be more inclusive. Given that age limits did not have an obvious association with infant homicide rates, the results are expected to be similar if these changes in age limit were accounted for.

Although infants make up a small percentage of homicide victims, these deaths are preventable. Programs and policies that strengthen economic supports for families, provide quality and affordable childcare, develop parenting skills (e.g., through home visiting programs), assure safe, stable, nurturing relationships and environments for all infants (*10*), and increase the public’s awareness of Safe Haven Laws might contribute to preventing infant homicides.

**Summary**

**What is already known about this topic?**

The highest risk for infant homicide is on the day of birth. Because of this, by 2008, all 50 states and Puerto Rico had enacted Safe Haven Laws to address infant abandonment and endangerment.

**What is added by this report?**

The infant homicide rate on the day of birth decreased from 222.2 per 100,000 person-years during 1989–1998 to 74.0 during 2008–2017 (66.7% decline) but remains at least 5.4 times higher than the rate at any other time in life.

**What are the implications for public health practice?**

Programs and policies that strengthen economic supports, provide affordable childcare, and enhance and improve skills for young parents might contribute to the prevention of infant homicides.

## Footnotes

* The District of Columbia did not enact Safe Haven Laws until 2009.

† Per the information contained in state Safe Haven Laws through December 2016, the specifics about who may legally surrender an infant under Safe Haven Laws vary by state. For example, in most states, either parent of the infant may legally surrender the infant to a safe haven. However, some states only allow the mother to surrender an infant to a safe haven, while other states allow a representative of the parent (person who has permission by the parent) to legally relinquish an infant to a safe haven. Other states specify who may legally surrender an infant under Safe Haven Laws in their state-specific statutes. https://www.childwelfare.gov/pubpdfs/safehaven.pdf.

§ Source: restricted-use National Vital Statistics System, linked birth and infant death data.

¶ ICD-10 codes U01 and U02 are codes for terrorism only used in the United States and are not a part of the ICD-10 underlying cause codes.

** This report used age limits of legal relinquishment specified in state-specific Safe Haven Laws as of December 2016. Any changes made to state-specific Safe Haven age limits after December 2016 are not accounted for in this report.

†† Homicide rates for infants (i.e., children aged <1 year) are commonly reported per 1,000 live births over a specified period, but alternatively, they can be reported per person-years. In this study, because actual time at risk for homicide is the outcome of interest, and infant homicides occurred during different times of infancy (e.g., day of birth, week one, week two, etc.), presenting rates in person-years allows for the calculation of homicide risk by week during infancy and by day during the first week of life.
